# Hydroxychloroquine induced lung cancer suppression by enhancing chemo-sensitization and promoting the transition of M2-TAMs to M1-like macrophages

**DOI:** 10.1186/s13046-018-0938-5

**Published:** 2018-10-29

**Authors:** Yong Li, Fengjun Cao, Mingxing Li, Pindong Li, Yuandong Yu, Longchao Xiang, Tao Xu, Jinhua Lei, Yun Yan Tai, Jianyong Zhu, Bingbing Yang, Yingpin Jiang, Xiufang Zhang, Long Duo, Ping Chen, Xiongjie Yu

**Affiliations:** 1 Department of Oncology, Renmin Hospital, Hubei University of Medicine, 39 Chaoyang middle Rd, Shiyan, 442000 Hubei China; 2 Department of Rehabilitation Medicine, Tongji Hospital, Tongji Medical College, Huazhong University of Science and Technology, Wuhan, 430030 China; 3 Cancer Center of Union Hospital, Tongji Medical College, Huazhong University of Science and Technology, Wuhan, 430022 China; 4 Department of Biochemistry and Molecular Biology, Tongji Medical College, Huazhong University of Science and Technology, Wuhan, 430030 China; 5 Department of Respiratory Medicine, Renmin Hospital, Hubei University of Medicine, Shiyan, 442000 China; 6 Teaching practice base of Oncology, Shiyan Renmin Hospital, Jinzhou Medical University, Shiyan, 442000 China; 7 Institute of Cancer Research, Renmin Hospital, Hubei University of Medicine, Shiyan, 442000 China

**Keywords:** Hydroxychloroquine, Lung cancer, Chemotherapy resistance, Lysosome, Acidification, Macrophage remodelling

## Abstract

**Background:**

Lysosome-associated agents have been implicated as possible chemo-sensitizers and immune regulators for cancer chemotherapy. We investigated the potential roles and mechanisms of hydroxychloroquine (HCQ) in combination with chemotherapy in lung cancer treatment.

**Methods:**

The effects of combined treatment on non-small cell lung cancer (NSCLC) were investigated using cell viability assays and animal models. The influence of HCQ on lysosomal pH was evaluated by lysosomal sensors and confocal microscopy. The effects of HCQ on the tumour immune microenvironment were analysed by flow cytometry.

**Results:**

HCQ elevates the lysosomal pH of cancer cells to inactivate P-gp while increasing drug release from the lysosome into the nucleus. Furthermore, single HCQ therapy inhibits lung cancer by inducing macrophage-modulated anti-tumour CD8^+^ T cell immunity. Moreover, HCQ could promote the transition of M2 tumour-associated macrophages (TAMs) into M1-like macrophages, leading to CD8^+^ T cell infiltration into the tumour microenvironment.

**Conclusions:**

HCQ exerts anti-NSCLC cells effects by reversing the drug sequestration in lysosomes and enhancing the CD8^+^ T cell immune response. These findings suggest that HCQ could act as a promising chemo-sensitizer and immune regulator for lung cancer chemotherapy in the clinic.

**Electronic supplementary material:**

The online version of this article (10.1186/s13046-018-0938-5) contains supplementary material, which is available to authorized users.

## Background

Lung cancer has recently become the third leading cause of cancer-related death worldwide [1, 2]. Approximately half of all non-small cell lung cancer (NSCLC) cases and nearly 80% of lung cancer cases are diagnosed at an advanced stage and have a poor prognosis after clinical treatment [3, 4]. Chemotherapy has been the standard therapy for NSCLC treatment in the clinic [2]. Unfortunately, traditional chemotherapeutic agents, such as mitomycin C (MMC), paclitaxel (PTX), cisplatin (Cis) and doxorubicin (DOX), have limited tumour-inhibiting effects and are often accompanied by severe side effects, including nausea, vomiting, hair loss, fatigue and diarrhea. Moreover, residual tumour cells and systemic toxicity associated with chemotherapy can exacerbate the host immune suppression, resulting in the aggravation of tumour progression. Thus, an innovative approach to enhancing the chemo-sensitivity of NSCLC cells to chemotherapy while efficiently eliminating immunosuppression is urgently needed in the clinic.

The addition of agents to enhance the efficacy of cancer therapy is a feasible strategy for decreasing the systemic effects of chemotherapy and improving outcomes. So far, lysosomes have been widely studied in tumour chemo-resistance [5–7]. One of the most common chemo-sensitizing methods is the application of agents to target tumour cell lysosomes [8]. Initially, the lysosome was reported to be an intracellular organelle that breaks down macromolecules and is involved in a variety of cellular processes. Recently, accumulating evidence has shown that the lysosomes can sequester drugs by over-expressing P-gp and subsequently digest extraneous chemotherapeutic agents via their acidic pH [9]. Drugs that accumulated in lysosomes in cancer cells loses efficacy, making the lysosome a potential target for enhancing sensitivity. In addition to target cancer cells directly, applying specific agents that induce host immune defences to suppress tumour growth may improve therapeutic outcomes. Recently, lysosome-associated agents, such as chloroquine (CQ), have been implicated as chemo-sensitizers [10, 11]. In fact, several clinical trials have evaluated the ability of CQ to enhance the efficacy of chemotherapy in a variety of cancer treatments (NCT00969306, NCT02432417) [12–14]. Moreover, CQ has been widely studied as an immune modulator in macrophages [15, 16]. However, in addition to its effectiveness in sensitizing tumour cells via chemotherapy, CQ was reported to induce severe side effects [17]. The identification of new agents that target lysosomes without inducing organ damage is urgently needed.

As a hydroxylation compound of CQ, hydroxychloroquine (HCQ) should be more suitable for impacting the lysosomal pH in cancer cells and potentially mediating anti-tumour immunity. However, the role of HCQ in NSCLC remains undefined. In the present study, we aimed to investigate the ability of HCQ to enhance chemotherapy sensitivity in NSCLC in vivo and in vitro, and we studied the potential mechanism of HCQ in chemo-sensitization. Here, we used an orthotopic mouse model for chemotherapy and HCQ treatment. Moreover, we assessed the immunomodulation by HCQ in the tumour microenvironment, including its effects on tumour-associated macrophages (TAMs) and CD8^+^ T cell in vivo and in vitro. We reasoned that elucidating the mechanism by which HCQ sensitizes NSCLC cells to chemotherapy and improving anti-tumour immunity could provide effective therapeutic strategies in the clinic.

## Methods

### Cell lines and reagents

Human non-small cell lung cancer cell line A549, murine lung cancer cell line Lewis and A549/adr cell line were purchased from Chinese Academy of Sciences Shanghai Cell Bank (Shanghai, China), and were culture in DMEM (Corning) supplemented with 10% FBS, 1% penicillin-streptomycin. Mitomycin C (MMC), paclitaxel (PTX), cisplatin (Cis), doxorubicin (DOX), NH_4_Cl and Methylamine (Met) were purchased from Sigma (USA). Verapamil was purchased from APExBIO (USA). MTT assay kit was purchased from Solarbio (China). Glutamic-pyruvic transaminase (GPT) assay kit, glutamic-oxaloacetic transaminase (GOT) assay kit and creatinine (CRE) assay kit were purchased from Solarbio (China).

### Animal studies

Female C57BL/6 J mice (4–6 weeks old) and BALB/c Nude Mice (4–6 weeks old) were purchased from the HFK Bio-Technology. Co., LTD (Beijing, China) and Vital River Laboratory Animal Technology Co., LTD (Beijing, China), respectively. To detect potential organ damage induced by HCQ, normal C57BL/6 J mice were used. After being randomly divided into two groups (5 mice per group), the mice were treated with either PBS or HCQ (10 mg/kg) by lung perfusion every 2 days for 2 weeks. Body weight was measured for all mice every 2 days, and after 14 days, the mice were sacrificed to obtain venous blood. Then, we examined the GOT/GPT/CRE level to evaluate organ damage. For all experiments involving the mouse tumour model, cells were intratracheally instilled into the lung. To evaluate the efficiency of HCQ, C57BL/6 J mice bearing Lewis lung tumours or nude mice bearing A549 lung tumours via intratracheal instillation were randomly divided into groups on day 5. As a control, mice were treated with PBS by tail vein injection from day 10 (Lewis-bearing mice) or day 20 (A549-bearing mice). In the chemotherapy groups, the mice were treated with DOX/MMC/PTX/Cis by tail vein injection every 2 days. In the HCQ groups, the mice were treated with 10 mg/kg HCQ by lung perfusion every 2 days. In the combination therapy group, the mice were treated with 10 mg/kg HCQ by lung perfusion, followed by DOX/MMC/PTX/Cis by tail vein injection every 2 days. The treatment was performed for 1 week. After 2 weeks, 5 mice from each group (the PBS group, HCQ group, chemotherapy group and HCQ combined with chemotherapy group) were sacrificed, and the lung weight was analysed to evaluate the anticancer effects. After gently washing with PBS, the samples were fixed in formalin, followed by embedding in paraffin for pathology and further studies. Another 10 mice in each group were used to record the survival time. For some experiments, the Lewis-bearing C57BL/6 J mouse model was also used to study the role of CD8^+^ T cells or tumour-associated macrophages, and neutralizing anti-CD8 antibodies, clodronate liposomes or neutralizing anti-CCL2 antibodies were intraperitoneally injected every 4 days. The groups and treatments were the same as the above with the addition of the neutralizing anti-CD8 antibody, clodronate liposome or neutralizing anti-CCL2 antibody treatment.

To determine whether the immune system participated in the HCQ-induced chemotherapy enhancement, the nude mouse model bearing Lewis/A549 cells was used. The groups were the same as those described above.

### RNA interference

A549 and Lewis cells (2 × 10^5^) were seeded onto 6-well plates and cultured at 37 °C. After 24 h, cells were transfected with siRNA using Lipofectamine 2000 (Invitrogen) following the manufacturer’s protocol. The sequences of siRNA were as follows: siRNA against human *Tfeb* (5’-CAGGCUGUCAUGCAUUACATT-3′ or 5’-GCGGCAGAAGAAAGACAAUTT-3′) and mice *Tfeb* (5’-TGTCTAGCAGCCACCTGAACGTGTA-3′ or 5’-ACAGTCCCATGGCCATGCTACATAT-3′).

### Cell viability assay

MTT assay was performed to detect the cell viability of A549, Lewis or A549/adr cells under different conditioned culture systems following the manufacturer’s protocol.

### H&E and immunohistochemistry

After sacrificing the mice, samples were fixed in 4% paraformaldehyde overnight and then embedded in paraffin. Paraffin sections were cut into 3 μm on a slicer (Leica RM2235, German). After dewaxing in xylene and gradient alcohol, the sections were then processed for H&E staining (Solarbio, China). Pictures were taken at a 20x (H&E) magnification by using a microscope (Leica DM3000, German). For the immunohistochemistry of CD8 in lung tumour tissues, antigen retrieval was performed by using citric acid and sodium citrate. Then the sections were incubated with CD8 (1:500, Abcam, USA) at 4 °C overnight and followed by signal amplification using a ABC HRP Kit (Thermo, USA). Microscope (Leica, German) was used to visualize the sections.

### Confocal microscopy

To illustrate role of lysosomes in sensitizing tumor cells, wild type or knocking down TFEB A549 cells were incubated with DOX at 37 °C for different periods of time after pre-treated with/without HCQ. After fixed and permeabilized, the cells were blocked with 5% BSA/PBS and incubated with primary antibody against LAMP2 (1:200, Abcam, USA), P-gp (1:100, Abcam, USA) and lysosome sensor (1:1000, Thermo, USA). Sections were then incubated with fluorescence-labeled secondary antibody (Life Technologies, USA), followed by counterstaining with DAPI (Invitrogen, USA). Images were captured with a confocal microscope (Olympus FV1000, Japan).

### qRT-PCR

Total RNA was isolated from cells under different conditioned culture systems. Then cDNA was synthesized using reversed transcriptional kit (Toyobo, Japan). Real-time PCR was performed on the Applied Biosystems Real-Time PCR cycler (Thermo Fisher, USA) with Fast SYBR Green PCR master mix (TOYOBO). The mRNA levels were normalized to β-actin. The primer pairs used were listed as follows: Human *Tfeb* sense:5’-CCTGGAGATGACCAACAAGCAG-3′, antisense: 5’-TAGGCAGCTCCTGCTTCACCAC-3′; Human *β-actin* sense: 5’-GCACCACACCTTCTACAATGAG- 3′, anti-sense: 5’-GGTCTCAAACATGATCTGGGTC-3′; Mouse *Tfeb* sense: 5′- GCTCCAACCCCGAGAAAGAG-3′, anti-sense: 5′- CAGCGTGTTAGGCATCTGC -3′; Mouse *Ifng* sense: 5’-GAGCCAGATTATCTCTTTCTACCT-3′, anti-sense: 5′- GTTGTTGACCTCAAACTTGGC-3′; Mouse *Tgfb1* sense: 5’-AACAATTCCTGGCGTTACCT-3′, anti-sense: 5’-GGCTGATCCCGTTGATTTCC-3′; Mouse *IL10* sense: 5’-CGGGAAGACAATAACTGCACCC-3′, anti-sense: 5’-CGGTTAGCAGTATGTT GTCCAGC-3′; Mouse *IL12b* sense: 5’-TGGTTTGCCATCGTTTTGCTG-3′, anti- sense: 5’-ACAGGTGAGGTTCACTGTTTCT-3′; Mouse *IL1b* sense: 5’-TGGACCTTCCAGGATGAGGACA-3′,anti-sense:5’-GTTCATCTCGGAGCCTGTAGTG-3’;Mouse *IL6* sense: 5’-TACCACTTCACAAGTCGGAGGC -3′,anti-sense: 5’-CTGCA AGTGCATCATCGTTGTTC-3′; Mouse *Vegfa* sense: 5’-CTGCTGTAACGATGAA GCCCTG-3′,anti-sense: 5’-GCTGTAGGAAGCTCATCTCTCC-3′; Mouse *Nos2* sense: 5’-GATGTTGAACTATGTCCTATCTCC-3′, anti-sense: 5’-GAACACCACTTTCACCAAGAC-3′; Mouse *Arg1* sense: 5’-CAAGACAGGGCTCCTTTCAG-3′, anti-sense: 5’-TGGCTTATGGTTACCCTCCC-3′; Mouse *Ido1* sense: 5′-GAGGATGCGTGACTTTGTGG-3′, anti-sense: 5’-ATCAAGACTCTGGAAGATGCTG-3′; Mouse *β-actin* sense: 5’-TTCCTTCTTGGGTATGGAATCCT-3′,anti- sense: 5′- CACTGTGTTGGCATAGAGGTC-3′.

### Lysosomal pH detection assay

Using Intracellular pH Calibration Buffer Kit, the lysosomal pH of Lewis and A549 cells under different condition systems were detected as previously reported [16]. Briefly, after washing Lewis and A549 cells with Live Cell Imaging Solution (LCIS), ¼ LCIS was replaced with the 1 mM Cell Loading Solution with Valinomycin/Nigericin and was incubated at 37 °C for 5 min. Then, the samples were analyzed using appropriate Ex/Em maxima. We also used lysosomal sensor to analyze the lysosomal pH influence by Confocal. Briefly, Lewis and A549 cells were pretreated with HCQ (5 μM, 12 h), then 1 mM Lyso-Sensor was added into the culture system. After 30 min, the cells were analyzed with a confocal microscope (Olympus FV1000, Japan).

### Tumor-infiltrating leucocytes isolation

Tumor nodules isolated from lung of Lewis-bearing mice were cut into small pieces. With 1 mg/ml collagenase (Sigma-Aldrich), 2 units/ml hyaluronidase (Sigma-Aldrich), and 0.1 mg/ml DNase (Sigma-Aldrich) digestion for 1 h, single cell suspension was centrifuged with Ficoll to collect Tumor-infiltrating leucocytes. In some cases, anti-mouse CD8 or anti-mouse F4/80 biotin were used to sorting tumor-derived CD8^+^ T cells or TAMs by Miltenyi Biotec separators respectively.

### T cell proliferation assay

For T cell proliferation by CSFE staining, CD8^+^ T cells were sorted from spleen single-cell suspensions by Miltenyi Biotec separators and stained with CFSE. Cells were incubated with IL-2 (R&D) and mouse CD3/CD28 Dynabeads (Thermo, USA) stimulation for 3 days, the HCQ treated or not CD8^+^ T cells were collected for Flow Cytometry analyses. For tumour-derived CD8^+^ T cell proliferation, the CD8^+^ T cells were sorted from tumour single-cell suspensions by Miltenyi Biotec separators. Cells were cultured in RPMI-1640 supplemented 10% FBS with or without IL-2 and/or CD3/CD28 beads stimulation. Three days later, the total cell number were counted.

### Mouse NK cell isolation and culture

To get the CD3^−^CD49b^+^ NK cells, CD3 negative cells were sorted from spleen single-cell suspensions by Miltenyi Biotec separators firstly. Then, the CD49b positive cells were sorted from the CD3 negative cells. NK cells were cultured in RPMI-1640 medium with 10% FBS, 1% L-Glutamine, 1% penicillin-streptomycin, 50 μM beta-mercaptoethanol and 10 ng/mL IL-2. Three days later, the supernatant with or without HCQ treatment was collected for enzyme-linked immunosorbent assay (ELISA). Mouse IFN-γ, TNF-α, IL-10 and MCP-1 ELISA kits were purchased from Dakewe Bioengineering (Shenzhen, China).

### Flow cytometry

Single cell suspension in PBS were stained with anti-mouse CD69 (H1.2F3), CD137 (17B5), CD3 (17A2), CD8a (53–6.7), CD206 (C068C2) and MHC II (M5/114.15.2) antibody at 4 °C for 30 min. In some cases, anti-mouse CD16/32 (93) antibody was used to block nonspecific binding of immunoglobulin to macrophage Fc receptors. After washing with PBS, cells were collected by BD Accuri C6 and data were analyzed with FlowJo software.

### Statistical analysis

Results were expressed as mean values ± SEM and interpreted by repeated-measure analysis of variance. Log-rank (Mantel-Cox) Test was used to analyze long-term survival curve. Differences were considered to be statistically significant when the *P*-value was < 0.05.

## Results

### Pre-instillation of HCQ enhances the efficacy of chemotherapy in lung cancer

**Fig. 1:**
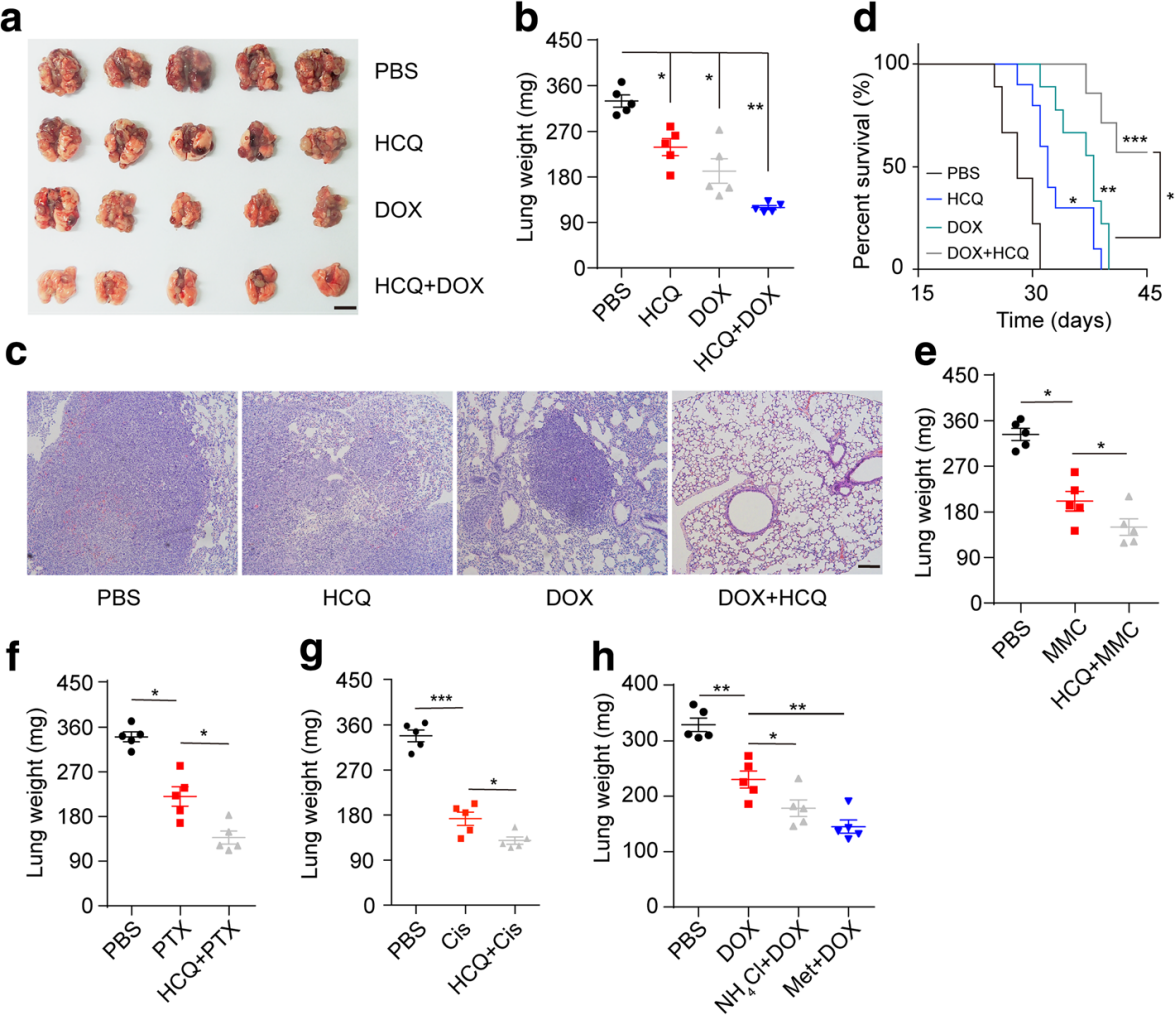
Pre-instillation of HCQ enhances the efficacy of chemotherapy in lung cancer. **a-d** A total of 2 × 10^5^ Lewis cells were intratracheally instilled into the lungs of C57BL/6 J mice. On day 10, mice were instilled with HCQ (10 mg/kg) by lung perfusion and then treated with DOX (2 mg/kg) treatment by tail vein injection every 2 days. The mice were treated for 1 week. After 2 weeks, 5 mice were sacrificed for tumour evaluation. **a** Representative images of the lungs in each treatment group. (scale bar, 5 mm) **b** Lung weight in each group (*n* = 5). **c** Histological H&E staining of the lung in each group (scale bar, 100 μm). **d** The long-term survival of tumour-bearing mice from each group (*n* = 10). **e** The lung weight of Lewis-bearing mice treated with PBS, MMC (2 mg/kg) and MMC combined with HCQ (*n* = 5). **f** The lung weight of Lewis-bearing mice treated with PBS, PTX (4 mg/kg) and PTX combined with HCQ (n = 5). **g** The lung weight of Lewis-bearing mice treated with PBS, Cis (4 mg/kg) and Cis combined with HCQ (n = 5). **h** The lung weight of Lewis-bearing mice treated with PBS, DOX (2 mg/kg), DOX combined with NH_4_Cl (10 mg/kg) and DOX combined with Met (10 mg/kg). For all graphs, the error bars indicate the mean ± s.e.m., *P < 0.05, **P < 0.01, ***P < 0.001. The data shown are representative of three independent experiments

Increasing evidence indicates that lysosome-associated agents might serve as chemo-sensitizers to enhance therapeutic effects. HCQ, a 4-amino-quinoline derivative and an anti-malarial drug, is reported to efficiently inhibit cellular lysosomal functions. To assess the potential application of HCQ in cancer therapy, an orthotopic lung cancer model was constructed by instilling cancer cells (A549 or Lewis) into the mouse lungs intratracheally. On day 10, the lungs of the mice were pre-instilled with HCQ, followed by a tail vein injection of DOX. After 2 weeks, there was a striking inhibition of tumour growth in the HCQ combined with DOX group, while both the HCQ and DOX groups showed anticancer effects compared with the PBS group in Lewis-bearing mice (Fig. 1a and b). Furthermore, haematoxylin and eosin (H&E) staining revealed that pre-instillation of HCQ before chemotherapy also efficiently suppressed the formation of pulmonary tumour nodules (Fig. 1c). Consistent with these findings, the survival time of the mice in the HCQ combined with DOX group was prolonged compared with that of the mice in the DOX alone treatment group (Fig. 1d). Additionally, compared with the DOX alone treatment, HCQ pre-instillation combined with DOX therapy decreased the lung weight in the A549 orthotopic lung cancer model (Additional file 1: Figure S1A). Other chemotherapeutic agents, such as MMC, PTX and Cis, also showed enhanced anticancer effects against lung cancer after the pre-instillation of HCQ (Fig. 1e-g). These results demonstrated that the application of HCQ could efficiently enhance the anticancer efficacy of chemotherapeutic agents for NSCLC. It has been reported that the inhibition of lysosomes in tumour cells might result in chemo-sensitization during cancer therapy. HCQ serves as a lysosome inhibitor and has been reported to be taken up by cellular lysosomes and to increase the lysosomal pH values. To investigate the correlation between lysosome inhibition and the sensitization effect caused by HCQ, we used two additional lysosome inhibitors, NH_4_Cl and methylamine (Met), to explore the enhanced anticancer effects caused by HCQ. Notably, both NH_4_Cl and Met enhanced the therapeutic efficiency of DOX (Fig. 1h), indicating that the sensitization effect involves lysosome inhibition in tumour cells and that several lysosome inhibitors have potency for chemo-sensitization in lung cancer therapy. In addition to the anticancer effects, the toxicity of an agent is an important indicator during the assessment of a treatment. In our study, the pre-instillation of HCQ induced neither hair nor weight changes in the mice (Additional file 1: Figure S1B). More importantly, no adverse effects on liver or kidney functions were observed in HCQ-treated mice (Additional file 1: Figure S1C-E). Together, these results reveal that HCQ efficiently potentiates the efficacy of chemotherapy in lung cancer treatment.

### HCQ enhances the sensitivity of lung cancer cells to chemotherapeutic drugs

**Fig. 2:**
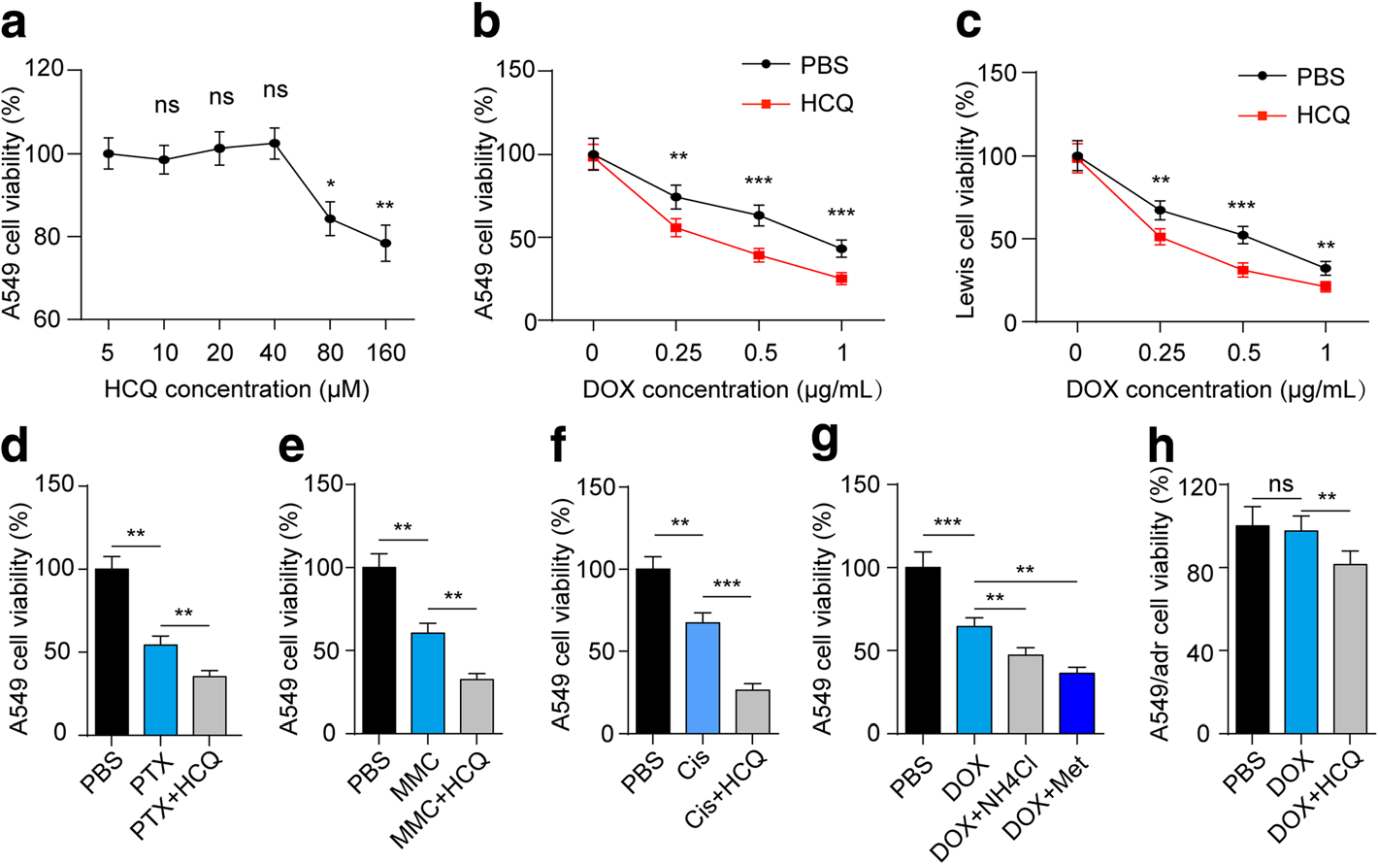
HCQ enhances the sensitivity of lung cancer cells to chemotherapeutic drugs. **a** A549 cells were treated with different concentrations of HCQ for 24 h. The cytotoxicity of HCQ was detected by MTT. **b** A549 cells were pre-treated with PBS or 5 μM HCQ, followed by DOX (0.5 μg/mL) treatment for 24 h. Cell viability was detected by MTT analysis. **c** Lewis cells were pre-treated with PBS or HCQ, followed by DOX treatment for 24 h. Cell viability was detected by MTT analysis. **d, e,** and **f** A549 cells were pre-treated with PBS or HCQ, followed by 1 μg/mL MMC, 0.5 μg/mL PTX or 0.5 μg/mL Cis treatment for 24 h. Cell viability was detected by MTT analysis. **g** A549 cells were treated with 1 μM NH_4_Cl or 1 μM Met, followed by DOX treatment for 24 h. Cell viability was detected by MTT analysis. **h** A549/adr cells were pre-treated with PBS or HCQ, followed by DOX (5 μg/ml) treatment for 24 h. Cell viability was detected by MTT analysis. For all graphs, the error bars indicate the mean ± s.e.m., *P < 0.05; **P < 0.01; ***P < 0.001; ns, no significant difference. The data shown are representative of three independent experiments

To further investigate the mechanism of the enhanced anticancer effects caused by HCQ, we hypothesized that HCQ might be cytotoxic to tumour cells and lead to cell proliferation inhibition. When the A549 cells were treated with HCQ at different concentrations, we observed the cytotoxic effects of HCQ only at high concentrations (> 80 μM) (Fig. 2a). However, the tumour cells that were pre-treated with HCQ at a low concentration (5 μM) showed enhanced sensitivity to DOX and were prone to death (Fig. 2b), even though HCQ caused no cytotoxicity. The same result was observed in Lewis cells (Fig. 2c), indicating that HCQ functions as a chemo-sensitizer to enhance the anticancer effects of chemotherapeutic agents without killing cancer cells directly. Moreover, other chemotherapeutic agents such as PTX, MMC and Cis also showed enhanced inhibition of tumour proliferation in A549 (Fig. 2d-f) and Lewis (Additional file 2: Figure S2A-C) cells subjected to HCQ pre-treatment. In addition to HCQ, different lysosome inhibitors (NH_4_Cl and Met) showed the same sensitizing effects on A549 (Fig. 2g) and Lewis (Additional file 2: Figure S2D) cells. Furthermore, the cytotoxicity of DOX was abolished in the A549/adr cell line (a DOX-resistant A549 cell line). However, DOX resulted in significant growth inhibition when the cells were pre-treated with HCQ (Fig. 2h). These results suggest that HCQ and the studied lysosome inhibitors enhance the cytotoxicity effects of chemotherapy.

### HCQ reverses drug sequestration in lysosomes by increasing the lysosomal pH to inactivate P-gp

**Fig. 3:**
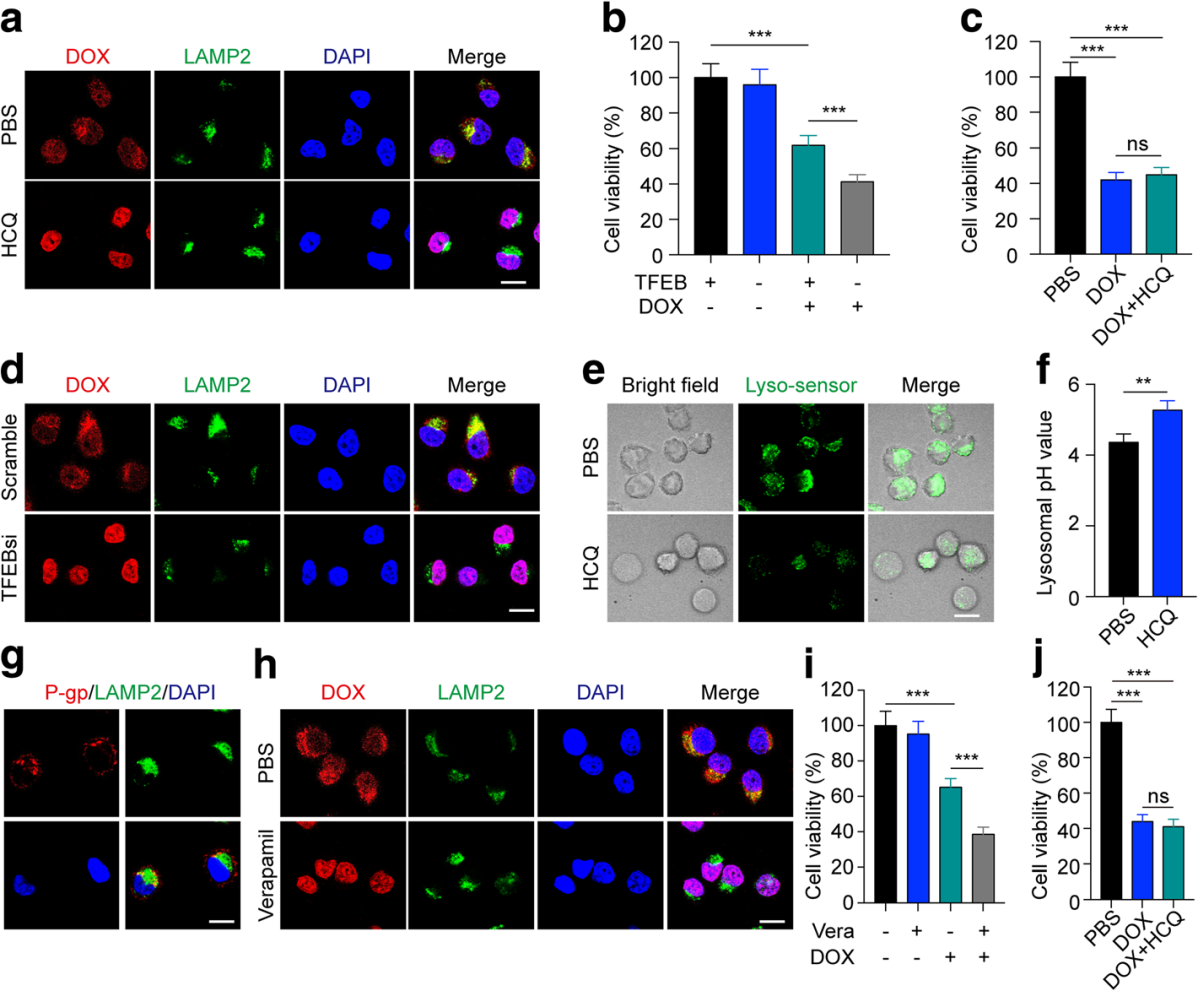
HCQ reverses drug sequestration in lysosomes by increasing the lysosomal pH to inactivate P-gp. **a** PBS- or HCQ-pre-treated A549 cells were treated with DOX (2 μg/ml, 2 h). The cells were stained with LAMP2 and analysed under a two-photon confocal microscope. Scale bar, 10 μm. **b** The viability of TFEB-silenced A549 cells treated with 0.5 μg/mL DOX for 24 h. **c** The viability of TFEB-silenced A549 cells treated with DOX or DOX combined with HCQ for 24 h. **d** TFEB-silenced (or not) A549 cells were treated with DOX (2 μg/ml, 2 h). The cells were stained with LAMP2 and analysed under a two-photon confocal microscope. Scale bar, 10 μm. **e** PBS- or HCQ-pre-treated A549 cells were treated with 1 μM lysosome sensor for 30 min and analysed under a two-photon confocal microscope. Scale bar, 10 μm. **f** The lysosomal pH values of A549 cells treated with PBS or HCQ for 12 h. **g** The A549/adr cells were stained with LAMP2, P-gp, and DAPI and analysed under a two-photon confocal microscope. Scale bar, 10 μm. **h** Verapamil-pre-treated (or not) A549/adr cells were treated with DOX (5 μg/ml, 2 h). The cells were stained with LAMP2 and analysed under a two-photon confocal microscope. Scale bar, 10 μm. **i** The viability of verapamil-pre-treated A549/adr cells treated with DOX for 24 h. **j** The viability of verapamil-pre-treated A549/adr cells treated with PBS, DOX or DOX combined with HCQ for 24 h. For all graphs, the error bars indicate the mean ± s.e.m., *P < 0.05; **P < 0.01; ***P < 0.001; ns, no significant difference. The data shown are representative of three independent experiments

Next, we sought to address the mechanism of HCQ sensitisation in lung cancer cells. Cytotoxicity is generally associated with the distribution of chemotherapeutic drugs in tumour cells. For example, many antineoplastic antimetabolites must enter the tumour cell nucleus and interfere with DNA function to ensure efficient killing of tumour cells. Herein, we traced the distribution of DOX in A549 cells by confocal microscopy. Interestingly, increased entry of DOX into HCQ pre-treated A549 cells was observed. More importantly, DOX accumulation in lysosomes was observed in the PBS group compared with the HCQ pre-treatment group (Fig. 3a). With respect to the lysosome inhibition effects of HCQ, we hypothesized that the sensitization caused by HCQ was induced by a lysosome-associated pathway. Here, we silenced the transcription factor EB (TFEB) (Additional file 3: Figure S3A and B), a master regulator of lysosomal biogenesis [18], and found that compared to the scramble group, the TFEB-silenced A549 cells showed enhanced sensitivity to DOX (Fig. 3b). However, HCQ failed to enhance the cytotoxicity of DOX in TFEB-knockdown A549 or Lewis cells (Fig. 3c and Additional file 3: Figure S3C), indicating that the NSCLC cell chemo-sensitization induced by HCQ is lysosome dependent. Furthermore, TFEB knockdown in A549 cells resulted in the increased entry of DOX into the nucleus and reduced accumulation of DOX in lysosomes, which was consistent with HCQ treatment (Fig. 3d). These results suggest that HCQ sensitizes the cancer cells to chemotherapeutic drugs through a lysosome-associated pathway.

The function of cellular lysosomes is to resolve the cellular debris and extraneous materials via hydrolase enzymes, which depend on the acidic lysosomal pH. Intriguingly, we observed that the lysosomal pH increased from 4.2 to 5.4 following treatment with HCQ in A549 cells (Fig. 3e and f) and Lewis cells (Additional file 3: Figure S3D). Previous studies have reported that the drug transporter P-glycoprotein (P-gp), a member of the ABC transporters, is localized in the lysosomal membrane and causes drug sequestration in lysosomes [19]. In addition, some lysosome inhibitors could reverse drug release from lysosomes, resulting in the sensitization of chemotherapeutic agents in tumour cells [9]. Here, we speculated that P-gp in tumour cell lysosomes might pump extraneous drugs from the cytoplasm into lysosomes, leading to the accumulation of drugs in lysosomes. In addition, pre-treatment with HCQ increases the lysosomal pH value, resulting in the functional inactivation of P-gp and drug release from lysosomes. Hence, P-gp-overexpressing A549/adr cells served as the model cells to detect the localization of P-gp. We observed the co-localization of P-gp and lysosome-associated membrane protein 2 (LAMP2) in A549/adr cells (Fig. 3g), indicating that P-gp was distributed in A549 cell lysosomes. Accordingly, pre-treatment with verapamil, an ABC transporter inhibitor, efficiently reversed the accumulation of DOX in lysosomes and increased DOX release from lysosomes to the nucleus (Fig. 3h). Moreover, the addition of verapamil sensitized the A549 cells to DOX (Fig. 3h and i), and the sensitizing effect of HCQ was eliminated in the presence of verapamil (Fig. 3j). The same results were observed in the A549/adr cell line (Additional file 3: Figure S3E and F), indicating that the HCQ-induced sensitization of the tumour cells is dependent on the activity of P-gp in lysosomes. Together, these results suggest that HCQ efficiently increases the lysosomal pH to interfere with the P-gp functions, leading to the reversal of drug sequestration in lysosomes.

### HCQ-induced tumour suppression is CD8^+^ T cell modulated

**Fig. 4:**
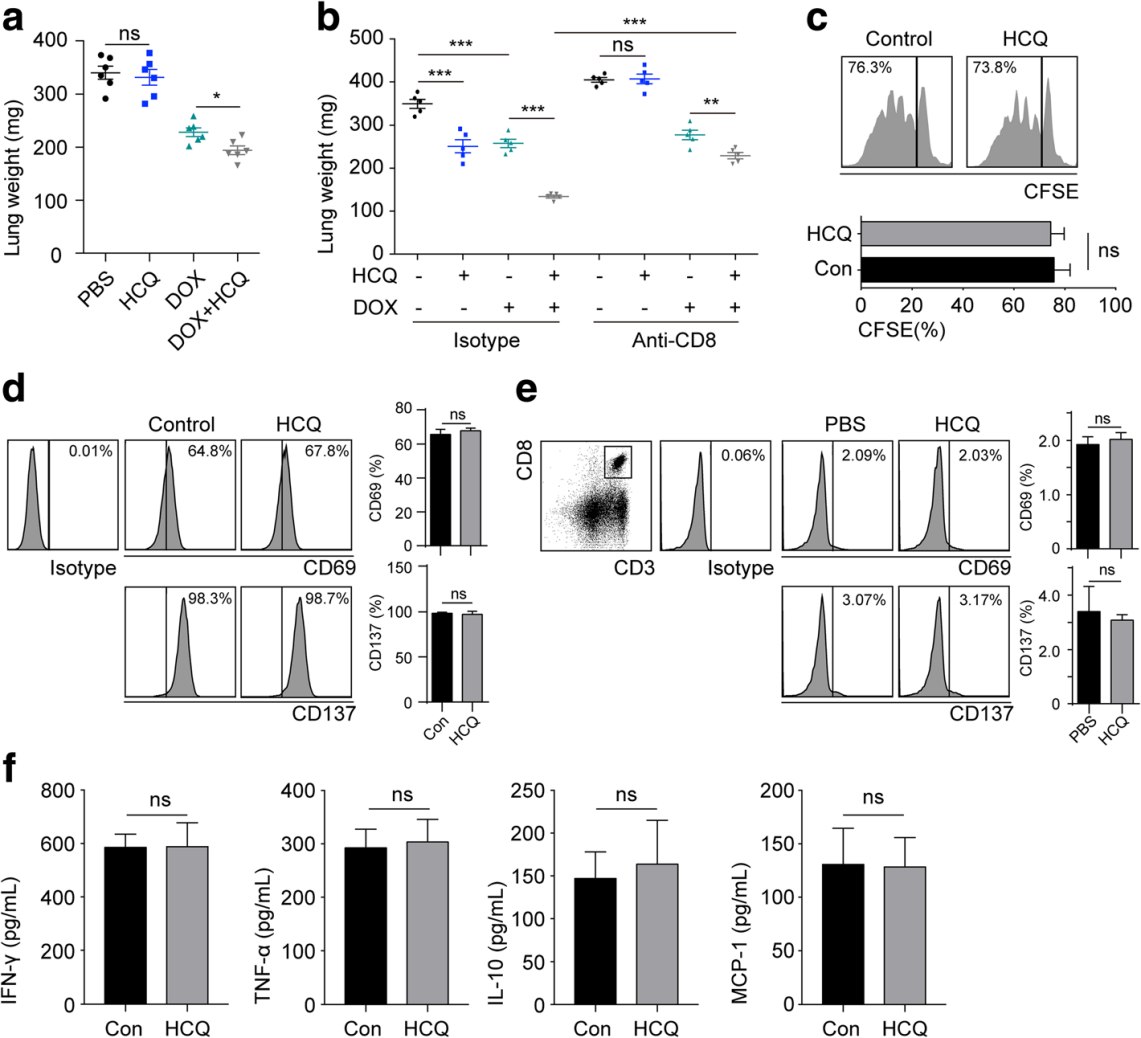
HCQ-induced tumour suppression is CD8^+^ T cell-modulated. **a** A total of 2 × 10^5^ Lewis cells were intratracheally instilled into the lungs of nude mice. On day 10, the mice were instilled with 10 mg/kg HCQ and/or followed with 2 mg/kg DOX treatment by tail vein injection. On day 24, the mice were sacrificed for lung weight evaluation (*n* = 6). **b** Lewis-bearing C57BL/6 J mice were instilled with 10 mg/kg HCQ and/or followed with i.v. 2 mg/kg DOX treatment in the presence or absence of isotype or anti-CD8 neutralizing antibody. On day 24, the mice were sacrificed for lung weight evaluation (*n* = 5). **c** CD8^+^ T cell isolated from the spleens of C57BL/6 J mice were labelled with CFSE and stimulated with CD3CD28 beads in the presence or absence of HCQ. The histogram of CFSE was analysed with flow cytometry after 72 h (*n* = 3). **d** CD8^+^ T cell isolated from the spleens of C57BL/6 J mice were treated with or without HCQ, and CD69 and CD137 expression was analysed by flow cytometry (n = 3). **e** Normal C57BL/6 J mice were treated with PBS or HCQ. CD69 and CD137 expression in CD3^+^CD8^+^ splenocytes were analysed by flow cytometry (n = 3). **f** CD3^−^CD49b^+^ NK cells were sorted from the spleen and treated with or without HCQ for 3 days. The level of IFN-γ, TNF-α, IL-10 and MCP-1 in supernatant were detected by ELISA (n = 3). For all graphs, the error bars indicate the mean ± s.e.m., *P < 0.05; **P < 0.01; ***P < 0.001; ns, no significant difference. The data shown are representative of three independent experiments

The above findings have addressed that HCQ uses the lysosomal pathway to enhance the efficacy of chemotherapy in lung cancer. Interestingly, mono-treatment with HCQ decreased the lung weight of Lewis cell tumours in immunocompetent C57BL/6 J mice (Fig. 1b). However, HCQ lost its anti-tumour ability in A549 tumours in nude mice (Additional file 1: Figure S1A). Although we have demonstrated that HCQ enhanced the chemotherapeutic efficacy by increasing the lysosome pH, there might be another factor contributing to the anti-tumour effect of HCQ. In addition to the sensitization effects caused by clinical agents, the immune microenvironment at the tumour site also plays a crucial role in tumour progression. The tumour immune response is mainly induced by specific T cells. Therefore, we used HCQ to treat Lewis-bearing nude mice (T cell-deficient) to explore the role of the T cell-induced anti-tumour immune response in NSCLC suppression. Intriguingly, HCQ failed to inhibit Lewis growth in the lung despite its chemo-sensitization with DOX, which is contrary to the result obtained in immunocompetent C57BL/6 J mice (Fig. 4a). These data demonstrated that T cells are required for the therapeutic effect of HCQ against lung cancer. The T cell-induced anti-tumour immune response is dependent on the CD8^+^ T cell. Therefore, we used anti-CD8 neutralizing antibodies to deplete CD8^+^ T cells in Lewis-bearing C57BL/6 J mice. We found that HCQ markedly decreased tumour weight in immunocompetent C57BL/6 J mice but did not show any influence in CD8^+^ T cell-depleted mice (Fig. 4b). These results suggest that HCQ-induced tumour suppression is CD8^+^ T cell-modulated. Then, we analysed the CD8^+^ T cell function with HCQ in vitro and in vivo. Interestingly, HCQ did not increase CD8^+^ T cell proliferation (Fig. 4c). Moreover, HCQ did not impact the activation of CD8^+^ T cell in vitro or in vivo, as evaluated by the expression of CD69 and CD137 (markers of the activated CD8^+^ T-cell) [20, 21] (Fig. 4d and e), suggesting that the anti-cancer effects induced by HCQ are mediated by CD8^+^ T cell rather than activating T cells directly. Additionally, natural killer (NK) cells play a critical role in the innate immune system, which provides a rapid response to viral infection and promotes tumour killing due to its nonspecific cytotoxic function. Here, to verify whether HCQ also modulated NK cell activation, we isolated CD3^−^CD49b^+^ NK cells from normal mouse spleens. When we treated NK cells with HCQ, the cytokine secretion of NK cells, including IFN-γ, TNF-α, IL-10 and MCP-1, did not change compared to that of the control group (Fig. 4f). These data suggest that HCQ did not induce NK cell activation or NK cell-associated anti-cancer effects. Taken together, the above data indicate that HCQ-mediated lung cancer suppression is CD8^+^ T cell-modulated, but HCQ cannot directly activate CD8^+^ T cell.

### HCQ induces CD8^+^ T cell-based tumour suppression via macrophages

**Fig. 5:**
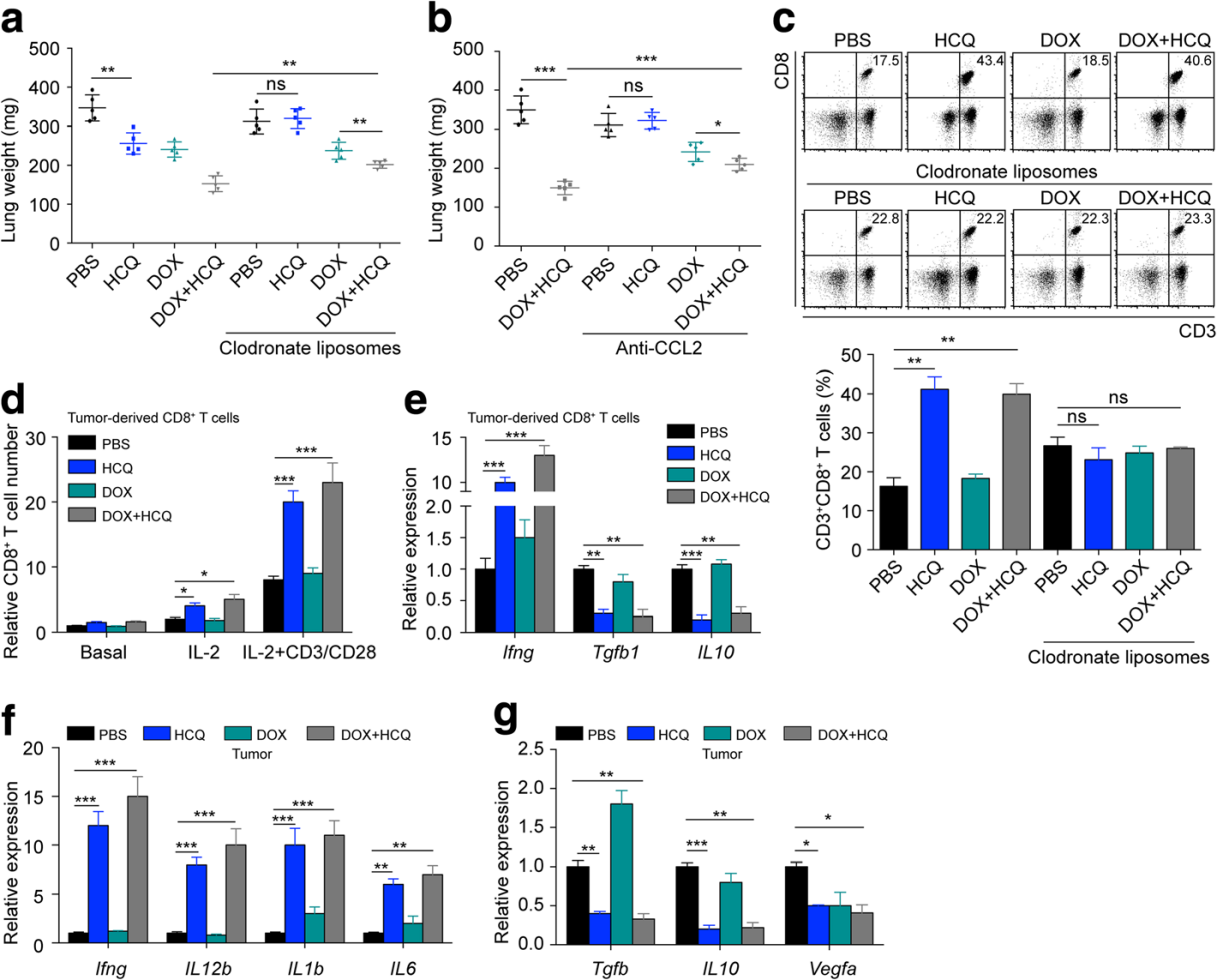
HCQ induces CD8^+^ T cell-based tumour suppression via macrophages. **a** Lewis-bearing C57BL/6 J mice (*n* = 5) were instilled with HCQ and/or i.v. followed by i.v. DOX treatment in the presence or absence of clodronate liposomes. On day 24, the mice were sacrificed for lung weight evaluation. **b** Lewis-bearing C57BL/6 J mice (*n* = 5) were instilled with HCQ and/or followed with i.v. DOX treatment in the presence or absence of anti-CCL2 antibody. On day 24, the mice were sacrificed for lung weight evaluation. **c** Tumour-infiltrating leukocytes (TILs) were isolated from (**a**), and the proportion of CD3^+^CD8^+^ T cell among TILs was analysed by flow cytometry (*n* = 3). **d** Tumour-derived CD8^+^ T cell were sorted from PBS- and HCQ-treated Lewis-bearing mice with or without additional DOX treatment and cultured with IL-2 or IL-2 + CD3/CD28 beads. The data were quantified by calculating the T cell number at day 3 compared with that at day 0 (*n* = 3). **e** The mRNA expression of *Ifng*, *Tgfb1* and *IL10* was analysed in tumour-derived CD8^+^ T cell, which were sorted from PBS- and HCQ-treated Lewis-bearing mice with or without additional DOX treatment (*n* = 3). **f** and **g** The mRNA expression of *Ifng*, *IL12b*, *IL1b*, *IL6*, *Tgfb1*, *IL10* and *Vegfa* was analysed in tumour tissue from PBS- and HCQ-treated Lewis-bearing mice in the presence or absence of DOX (*n* = 3). For all graphs, the error bars indicate the mean ± s.e.m., *P < 0.05; **P < 0.01; ***P < 0.001; ns, no significant difference. The data shown are representative of three independent experiments

Next, we wondered how HCQ exerted an anti-tumour effect on CD8^+^ T cell. In addition to the T cells in tumour tissues, many other leucocytes are infiltrated in the tumour microenvironment as well. Macrophages serve as the major component of innate immunity and heavily infiltrate the tumour microenvironment. To determine whether macrophages were involved in HCQ-induced tumour suppression, we used clodronate liposomes to deplete macrophages in mice. Intriguingly, we found that HCQ lost its tumour inhibition effect in lung cancer after macrophage depletion (Fig. 5a). Moreover, when an anti-CCL2 antibody was used to decrease macrophage infiltration in lung tumours, the tumour suppression mediated by HCQ was abolished as well (Fig. 5b). These data suggest that the HCQ-induced anti-tumour effect is macrophage-modulated. CD8^+^ T cell, also called cytotoxic T cells (CTLs), are crucial for anti-tumour immunity. Since both CD8^+^ T cell and macrophages are needed for HCQ-induced lung cancer inhibition, we analysed the connection between these cells in more depth. We detected the CD3^+^CD8^+^ T cell infiltration in Lewis-bearing immunocompetent or macrophage-depleted mice by flow cytometry and immunohistochemistry, and we found that HCQ increased CD3^+^CD8^+^ T cell numbers (Fig. 5c) and infiltration (Additional file 4: Figure S4A) with or without DOX treatment, but this phenomenon disappeared after macrophage depletion. These data suggest that HCQ-induced CD8^+^ T cell infiltration is macrophage-modulated. Next, we treated tumour bearing immunocompetent C57BL/6 J mice with HCQ in the presence or absence of DOX and sorted tumour-infiltrating CD8^+^ T cell from the orthotopic lung cancer model. We found that HCQ increased T cell proliferation with or without CD3/CD28 bead stimulation (Fig. 5d). Furthermore, HCQ up-regulated the expression of *Ifng* and down-regulated the mRNA levels of *Tgfb1* and *IL10* in tumour-infiltrating CD8^+^ T cell from tumour tissues (Fig. 5e). Additionally, we analysed the whole tumour immune microenvironment in HCQ- and/or DOX-treated mice with orthotopic lung cancer, and we found that HCQ with or without DOX treatment increased inflammatory cytokine expression, such as *Ifng*, *IL12b*, *IL1b* and *IL6* (Fig. 5f), and decreased *Tgfb1*, *IL10* and *Vegfa* expression (Fig. 5g). Together, these results reveal that HCQ induces CD8^+^ T cell-based tumour suppression via macrophages in vivo.

### HCQ induces the transition of M2-TAMs to M1-like macrophages

**Fig. 6:**
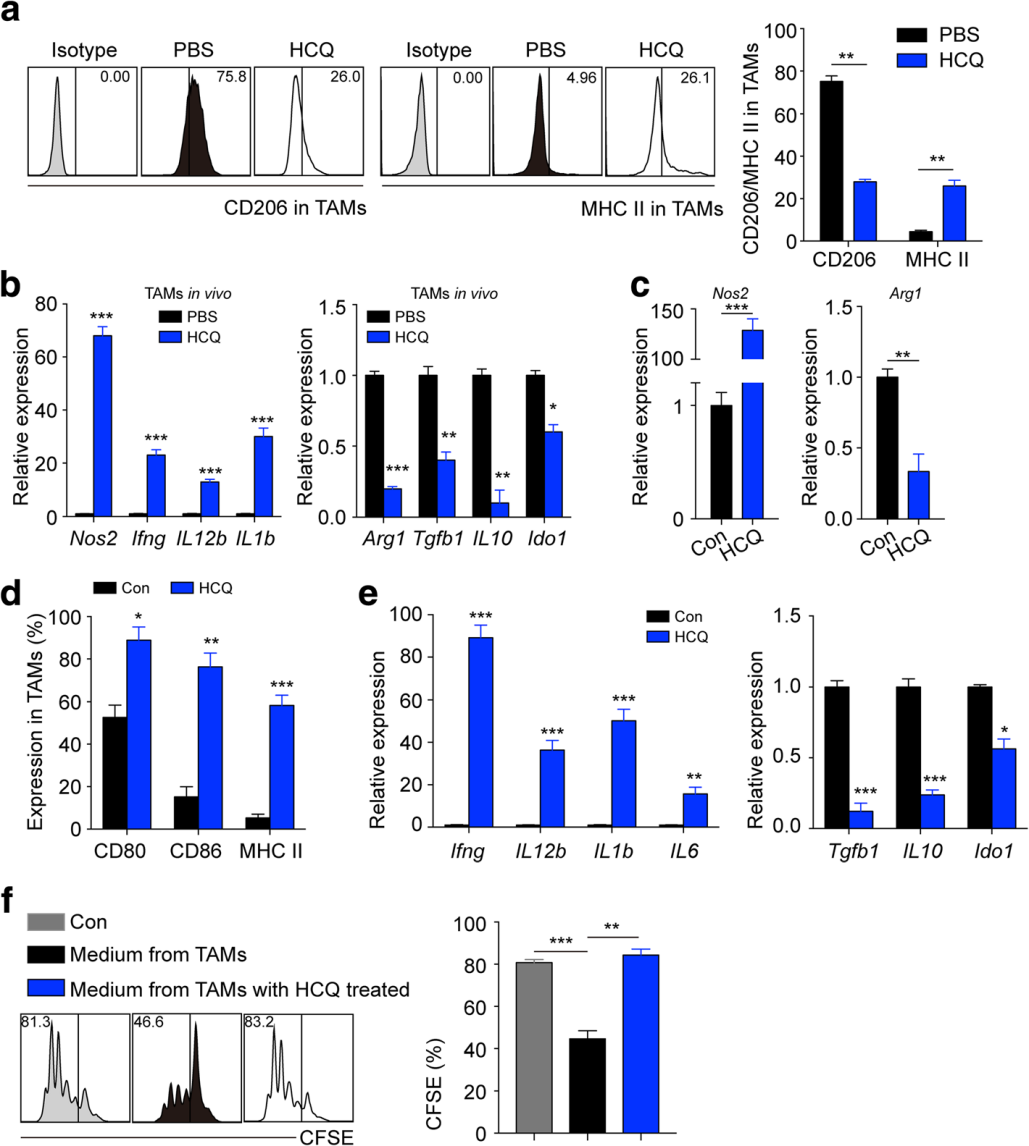
HCQ induces the transition of M2-TAMs to M1-like macrophages. **a** The representative flow cytometric histogram and quantification of CD206 and MHC II in TAMs (F4/80^+^ TILs), which were isolated from PBS- or HCQ-treated Lewis-bearing mice (*n* = 3). **b** TAMs were sorted from PBS- or HCQ-treated Lewis-bearing mice (n = 3), and the mRNA expression of *Nos2*, *Ifng*, *IL12b*, *IL1b*, *Arg1, Tgfb1*, *IL10* and *Ido1* was analysed. **c** TAMs were sorted from Lewis-bearing mice and treated with HCQ or not in vitro. The mRNA expression of *Nos2* and *Arg1* was analysed (*n* = 3). **d** Quantification of CD80, CD86 and MHC II in TAMs with or without HCQ treatment in vitro (*n* = 3). **e** TAMs were treated with HCQ or not in vitro. The mRNA expression of *Ifng*, *IL12b*, *IL1b*, *IL6*, *Tgfb1*, *IL10* and *Ido1* was analysed (*n* = 3). **f** TAMs were treated with HCQ or not, and the supernatant was collected to culture CFSE-labelled CD8^+^ T cell with CD3/CD28 bead stimulation. After 72 h, the histogram of CFSE was analysed by flow cytometry (*n* = 3). For all graphs, the error bars indicate the mean ± s.e.m., *P < 0.05; **P < 0.01; ***P < 0.001; ns, no significant difference. The data shown are representative of three independent experiments

Next, we investigated how HCQ mobilized macrophages to enhance CD8^+^ T cell anti-tumour immunity. Many studies have reported that macrophages were transitioned into tumour-associated macrophages (TAMs) by tumour cells or tumour secretions in the tumour microenvironment. TAMs are associated with tumour growth, angiogenesis, metastasis and immune escape [22, 23]. Thus, we hypothesized that HCQ may lead to the transformation of TAMs to induce anti-tumour T cell immunity in orthotopic lung cancer. We detected the phenotype of TAMs in the tumour microenvironment. Interestingly, we found that HCQ decreased the expression of CD206, which is an M2-like macrophage marker, but increased the expression of MHC II, which is an M1-like macrophage marker (Fig. 6a). Furthermore, we isolated F4/80^+^ TAMs from PBS- or HCQ-treated mice with orthotopic lung cancer, and we found that the M1-like molecules (*Nos2* [24]*, Ifng, IL12b,*and *IL1b*) were up-regulated and the M2-like molecules (*Arg1* [25]*, Tgfb1, IL10,* and *Ido1*) were down-regulated with HCQ treatment (Fig. 6b). This result suggested that HCQ induces TAMs from M2-like macrophages to become M1-like macrophages in vivo. Furthermore, TAMs were isolated from Lewis-bearing mouse tumour tissues and cultured with or without HCQ in vitro. We found that HCQ strongly induced *Nos2* up-regulation and *Arg1* inhibition (Fig. 6c). Additionally, HCQ enhanced costimulatory molecular expression, such as CD80, CD86, and MHC II (Fig. 6d). Moreover, HCQ-incubated TAMs showed increased M1-like cytokine (*Ifng, IL12b, IL1b,* and *IL6*) mRNA levels and decreased M2-like molecule (*Tgfb1, IL10,* and *Ido1*) expression (Fig. 6e). These data suggest that HCQ switches M2-like TAMs into M1-like macrophages in vitro. Furthermore, we collected the supernatant of TAMs with or without HCQ incubation for a CD8^+^ T cell proliferation assay. We found that the medium from TAMs inhibited CD3/CD28-stimulated CD8^+^ T cell proliferation, but medium from HCQ-incubated TAMs relieved this suppression (Fig. 6f). Taken together, these results indicate that HCQ switches M2-TAMs into M1-like macrophages to enhance CD8^+^ T cell immunity.

## Discussion

**Fig. 7:**
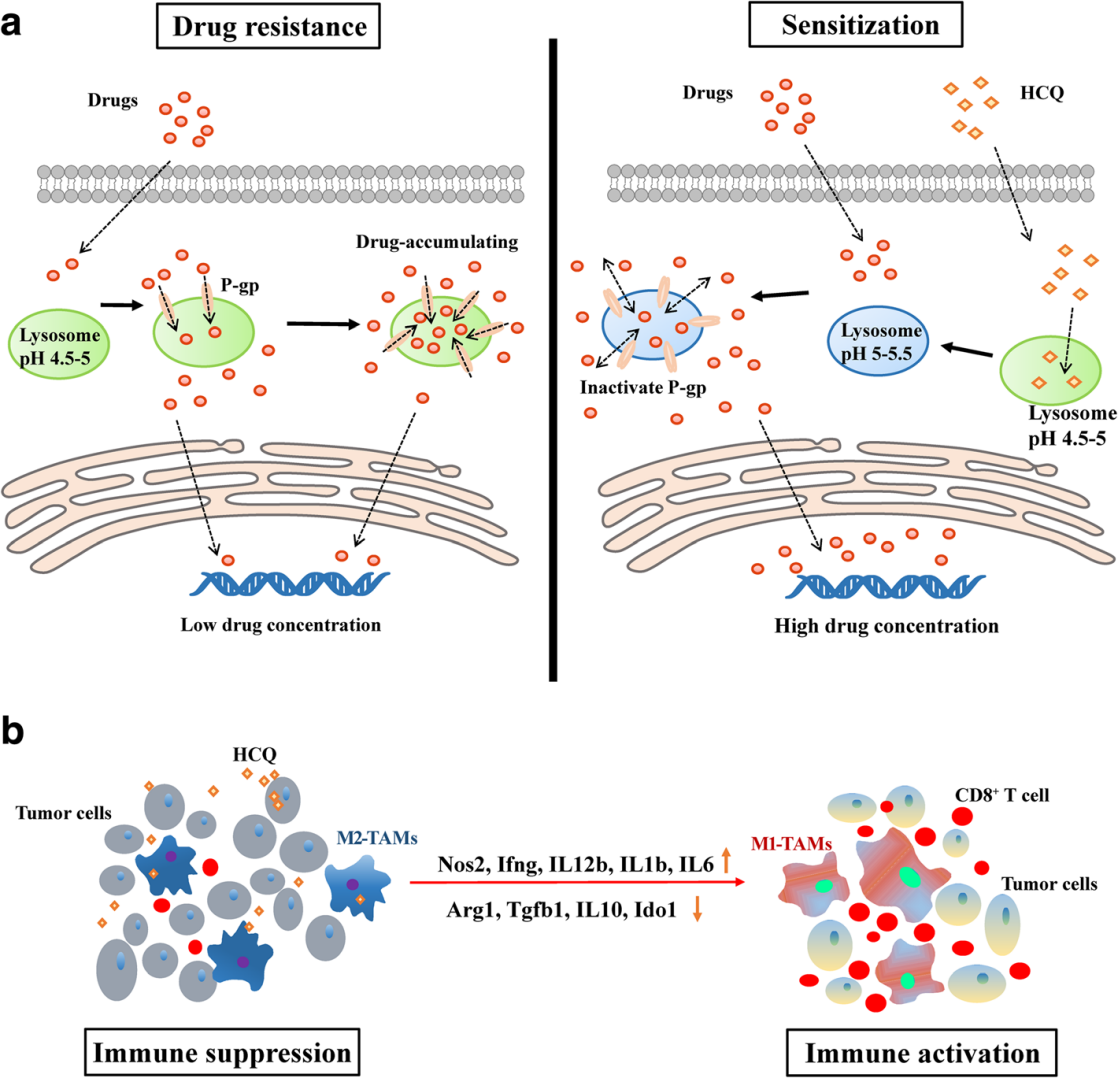
The schematic diagram of HCQ enhancing chemo-sensitization and switching M2-TAMs into M1-like macrophages. **a** HCQ increases lysosome pH of lung cancer cells and inactivates P-gp in lysosomes to endow drug release into the nucleus for chemo-sensitization. **b** HCQ up-regulates inflammatory cytokines and down-regulates immunosuppressive cytokines in TAMs to reverse M2-TAMs into M1-like macrophages and induce CD8^+^ T cell infiltration

In this study, we evaluated the ability of HCQ to increase the sensitivity of NSCLC cells to chemotherapeutic drugs both in vitro and in an established orthotopic mouse model. We found that HCQ, working as a chemo-enhancer, increased therapeutic efficacy in an immune-mediated manner. Moreover, we demonstrated that HCQ could reverse the specific role of lysosomes in sequestering chemotherapeutic agents through increasing the pH in lysosomes and could switch M2-TAMs into M1-like macrophages to induce CD8^+^ T cell infiltration into the tumour site (Fig. 7). These findings defined HCQ as a promising chemo-sensitizer and immune regulator with the potential to enhance the chemotherapeutic efficacy for NSCLC in clinical treatment.

Chemotherapy is the most widely used method of NSCLC treatments [26, 27]. However, accumulating evidence has shown that chemotherapeutic agents, such as DOX, MMC, Cis and PTX, can induce not only severe tumour inhibition but also side effects in a dose-dependent manner [28–30]. The efficacy of chemotherapeutic agents depends on several factors, especially the dosage of chemotherapeutic agents. Thereby, the development of therapies to increase the sensitivity of cancer cells and achieve superior efficacy with low-dose chemo-agents to avoid the systemic toxicity is urgently needed. Indeed, the administration of chemo-sensitizers has been reported to efficiently enhance the cytotoxicity of chemotherapy, resulting in prolonged survival time in mouse models [31, 32]. Similar results were achieved when we employed the lysosome inhibitors HCQ, NH4Cl and Met in combination with chemotherapy in this study. As a chemo-sensitizer, traditional lysosome inhibitors, such as CQ, showed a strong ability to increase the sensitivity of cancer cells to chemotherapy in a variety of cancers [32–34]. Nonetheless, unexpected side effects on organs such as the nephrotoxicity induced by CQ monotherapy were also observed, which is a reminder that this treatment is far from satisfactory [35, 36]. Thus, an analogue of chloroquine, HCQ, was synthesized to replace CQ to achieve superior anticancer effects and reduced organ damage. Like CQ, HCQ is involved in sensitizing cancer cells by decreasing lysosomal acidification, followed by the release of segregated chemotherapeutic drugs from lysosomes to the cytoplasm or nucleus, resulting in increased cytotoxicity of chemotherapy [37, 38]. Similarly, our data showed that HCQ significantly enhanced the efficacy of chemotherapy for NSCLC both in vitro and in vivo. Moreover, we found that HCQ alone had no side effects on NSCLC cell viability except at high concentrations, while it could sensitize cancer cells at concentrations as low as 5 μM, this finding demonstrates that sensitization effects occur at a low HCQ dose and result in reduced potential systemic toxicity. Most importantly, the lung perfusion of HCQ maximizes drug exposure at tumour sites while minimizing the drug distribution in the peripheral blood or normal tissues, resulting in a reduced risk of additional organ damage.

Since increasing attention has been paid to the potential application of lysosome inhibition in tumour therapy, chemo-sensitizers targeting lysosomes have been reported and even tested in clinical trials [32]. A phase I clinical trial (NCT01026844) that combined HCQ with erlotinib to treat advanced NSCLC in 2012 reported that HCQ was safe and well tolerated [39]. This trial suggested that HCQ was suitable for clinical application. However, their phase II clinical trial (NCT00977470) that combined HCQ with erlotinib for cancer treatment failed. Considering that erlotinib serves as an epidermal growth factor receptor (EGFR) inhibitor to target the tyrosine kinase domain in the cell membrane, erlotinib could not be absorbed by lysosomes, resulting in failed clinical treatment. This work suggested that the drugs combined with HCQ should be associated with the lysosome. The ability of lysosomes to sequester chemotherapeutic agents is often considered one of the explanations for the low sensitivity of cancer cells [31, 40]. It has been reported that P-gp (an ABC transporter) is over-expressed in lysosomes and contributes to lysosomal drug accumulation [41]. Our studies further demonstrated that the drug-accumulating lysosomes induced by P-gp could protect cancer cells from death caused by chemotherapeutic drugs. Therefore, it is necessary to inhibit the lysosomal function of sequestering drugs to enhance the efficacy of chemotherapy. In this study, the application of HCQ efficiently reversed the drug segregation effects of lysosomes and facilitated the accumulation of DOX in the nucleus, resulting in elevated cytotoxicity and improved anticancer effects.

Chemotherapy slows tumour progression and prolongs the patient survival time. However, it fails to eliminate all tumour cells in vivo, and the remaining tumour cells can educate the host tumour immune microenvironment to facilitate tumour recurrence and metastasis. Thus, there is an urgent need to find a compound that has synergistic effects when combined with chemotherapeutic agents to induce an anti-tumor immune response. Of note, HCQ is reported to play a role in the treatment of immune-mediated inflammatory disorders through impacts on the immune system [42]. In the present study, treatment of the nude mouse model bearing Lewis cells with HCQ/DOX failed to achieve a similar suppression of tumour growth. Considering the characteristics of nude mice, we hypothesized that the function of HCQ depended on T cells. In fact, the role of CD8^+^ T cell in cancer suppression has been investigated in many types of human malignancies, including breast cancer, oesophageal cancer, gastric cancer, pancreatic cancer, liver cancer, and colorectal cancer [43–47]. The majority of these studies indicated that patient prognosis is often directly correlated with the infiltrating CD8^+^ T cell number and activation state at the tumour sites. The data presented here verified the important role of CD8^+^ T cell in the HCQ-mediated anti-tumour effect. However, we observed that HCQ had no effect on CD8^+^ T cell proliferation or activation, which suggested that HCQ may function through other components of the immune system. Moreover, HCQ did not affect the activation of NK cells, indicating that other immune cells in the tumour microenvironment might participate in the immune activation induced by HCQ.

The tumour microenvironment (TME) comprises a variety of components, including tumour cells, cancer-associated fibroblasts, extracellular matrix, and TAMs [48, 49]. The TME provides support for tumour cells during the T cell-mediated anti-tumour immune response and clinical anticancer therapy [50]. One of the major protectors is TAMs [51]. TAMs have been reported to have substantial crosstalk with tumour cells and immune cells. When macrophages were eradicated in the NSCLC mouse model, we detected less infiltration of CD8^+^ T cell in tumour tissues. This finding proved that macrophages are required for in CD8^+^ T cell to induce the anti-tumour ability of HCQ. Of note, TAMs are classified into two major phenotypes, M1 and M2. While M1-TAMs are involved in suppressing cancer progression, M2-TAMs are inflammatory suppression and promote the tumour growth and invasion [52]. Considering the opposing roles of M1 and M2-TAMs at the tumour site, it is a prospect to block the M2-TAMs phenotype and its tumour-promoting behaviours to enhance the anticancer activity. In fact, several studies have proved that the process of converting M2 to M1 macrophages is feasible and therapeutic [53]. For example, using a CSF1R inhibitor (PLX3397 or BLZ945) to repolarize TAMs to the M1 type reduced tumour volume and prolonged survival time in both glioblastoma and breast cancer mouse models [54, 55]. Here, in our study, which investigated HCQ as a clinical drug, it is beneficial that HCQ is inexpensive and accessible. Our observations demonstrated that HCQ fostered the transition of M2 macrophages to M1 to enhance the anti-tumour effect of CD8^+^ T cell in NSCLC. Combined with chemotherapeutics, HCQ promoted the chemo-sensitization and killed the bulk of tumour cells. In addition to tumour mass reduction, killed tumour cells may release tumour antigens that can promote anti-tumour immunity. Further, HCQ reprogrammed M2-TAMs into M1 cells and recruited CD8^+^ T cell into tumour microenvironment to subject tumour cells to a second hit and induce more potent tumour-killing effects.

Compared with the reported articles, our study has unique advantages: (I) the chemo-sensitizer we used, HCQ, could effectively sensitize NSCLC cells at a low dose compared with those of traditional chemo-sensitizers; (II) HCQ had no toxicity on organs at the effective concentration and was safer and better tolerated in clinical therapy; (III) HCQ functioned not only through directly sensitizing NSCLC cells but also via fostering the transition of M2-TAMs to M1 macrophages that subsequently recruited and activated CD8^+^ T cell; and (IV) we used the orthotopic mouse model, which mimics the clinical drug delivery method, to evaluate whether this method is feasible in the clinic, which ensures maximal drug exposure to tumour cells and minimal drug circulation through the body. These advantages further strengthen the potential of HCQ as an ideal chemo-sensitizer and immune regulator for enhancing the therapeutic effects of NSCLC treatment.

## Conclusions

In summary, the data presented in this study clearly showed that by increasing the lysosomal pH in cancer cells and fostering the transition of M2-TAMs to M1 macrophages to induce CD8^+^ T cell anti-tumour effects, HCQ could function as a chemo-sensitizer and immune regulator to enhance the therapeutic effects of NSCLC. HCQ is a promising agent for cancer treatment to improve the quality of life of NSCLC patients.

## Additional files

Additional file 1: **Figure S1.** (A) A total of 2 × 10^6^ A549 cells were intratracheally instilled into the lungs of nude mice. On day 20, the mice were instilled with 10 mg/kg HCQ and then followed with 2 mg/kg DOX treatment by tail vein injection every 2 days. Mice were treated for a week. After 2 weeks, mice were sacrificed for lung weight evaluation. (B, C, D, E) Female C57BL/6 J mice were treated with 10 mg/kg HCQ. The mice were sacrificed on day 3 for the systemic toxicity analysis. (B) Weights of the mice in each group. (C) Glutamic-oxaloacetic transaminase (GOT) detection in each group. (D) Glutamic-pyruvic transaminase (GPT) detection in each group. (E) Creatinine (CRE) detection in each group. For all graphs, the error bars indicate the mean ± s.e.m., *P < 0.05; **P < 0.01; ***P < 0.001; ns, no significant difference. The data shown are representative of three independent experiments. (TIF 1095 kb)

Additional file 2: **Figure S2.** (A) Lewis cells were pre-treated with PBS or 5 μM HCQ and followed by 0.5 μg/mL PTX treatment for 24 h. Apoptosis was detected by MTT analysis. (B) Lewis cells were pre-treated with PBS or 5 μM HCQ and followed by 1 μg/mL MMC treatment for 24 h. Apoptosis was detected by MTT analysis. (C) Lewis cells were pre-treated with PBS or 5 μM HCQ and followed by 0.5 μg/mL Cis treatment for 24 h. Apoptosis was detected by MTT analysis. (D) A549 cells were treated with 1 μM NH_4_Cl or 1 μM Met and followed by 0.5 μg/mL DOX treatment for 24 h. Apoptosis was detected by MTT analysis. For all graphs, the error bars indicate the mean ± s.e.m., *P < 0.05; **P < 0.01; ***P < 0.001; ns, no significant difference. The data shown are representative of three independent experiments. (TIF 953 kb)

Additional file 3: **Figure S3.** (A) Relative TFEB expression in mRNA levels of A549 cells treated with TFEB siRNA. (B) Relative TFEB expression in mRNA levels of Lewis cells treated with TFEB siRNA. (C) The viability of TFEB-silenced Lewis cells treated with DOX or 0.5 μg/mL DOX combined with 5 μM HCQ for 24 h. (D) The lysosomal pH values of Lewis cells treated with PBS or HCQ (5 μM) for 12 h. (E) The viability of verapamil-pre-treated A549 cells treated with 0.5 μg/mL DOX for 24 h. (F) The viability of verapamil-pre-treated A549 cells treated with PBS, 0.5 μg/mL DOX or DOX combined with 5 μM HCQ for 24 h. For all graphs, the error bars indicate the mean ± s.e.m., *P < 0.05; **P < 0.01; ***P < 0.001; ns, no significant difference. The data shown are representative of three independent experiments. (TIF 1530 kb)

Additional file 4: **Figure S4.** (A) Lewis-bearing C57BL/6 J mice (*n* = 5) were instilled with HCQ and/or i.v. followed by i.v. DOX treatment in the presence or absence of clodronate liposomes. On day 24, mice were sacrificed and tumour tissues were fixed for immunohistochemistry. The represented pictures showed the CD8 expression of lung tumour tissues in each group (scale bar, 50 μm). (TIF 7658 kb)

## Abbreviations

ABC: ATP-binding cassette
CCL2: Chemokine (C-C motif) ligand 2
CTLs: Cytotoxic T cells
DOX: Doxorubicin
H&E: Hematoxylin and eosin
HCQ: Hydroxychloroquine
LAMP2: Lysosome-associated membrane protein 2
MMC: Mitomycin C
NSCLC: Non-small cell lung cancer
P-gp: P-glycoprotein
PTX: Paclitaxel
qRT-PCR: Quantitative reverse transcription polymerase chain reaction
siRNA: Small interfering RNA
TAMs: Tumour-associated macrophages
TEM: Tumour microenvironment
TFEB: Transcription factor EB
