# Supplementary figures and images for: Hydroxychloroquine induced lung cancer suppression by enhancing chemo-sensitization and promoting the transition of M2-TAMs to M1-like macrophages
Source: J Exp Clin Cancer Res. 2018 Oct 29;37:259. doi: 10.1186/s13046-018-0938-5 (PMC6206903; doi:10.1186/s13046-018-0938-5)

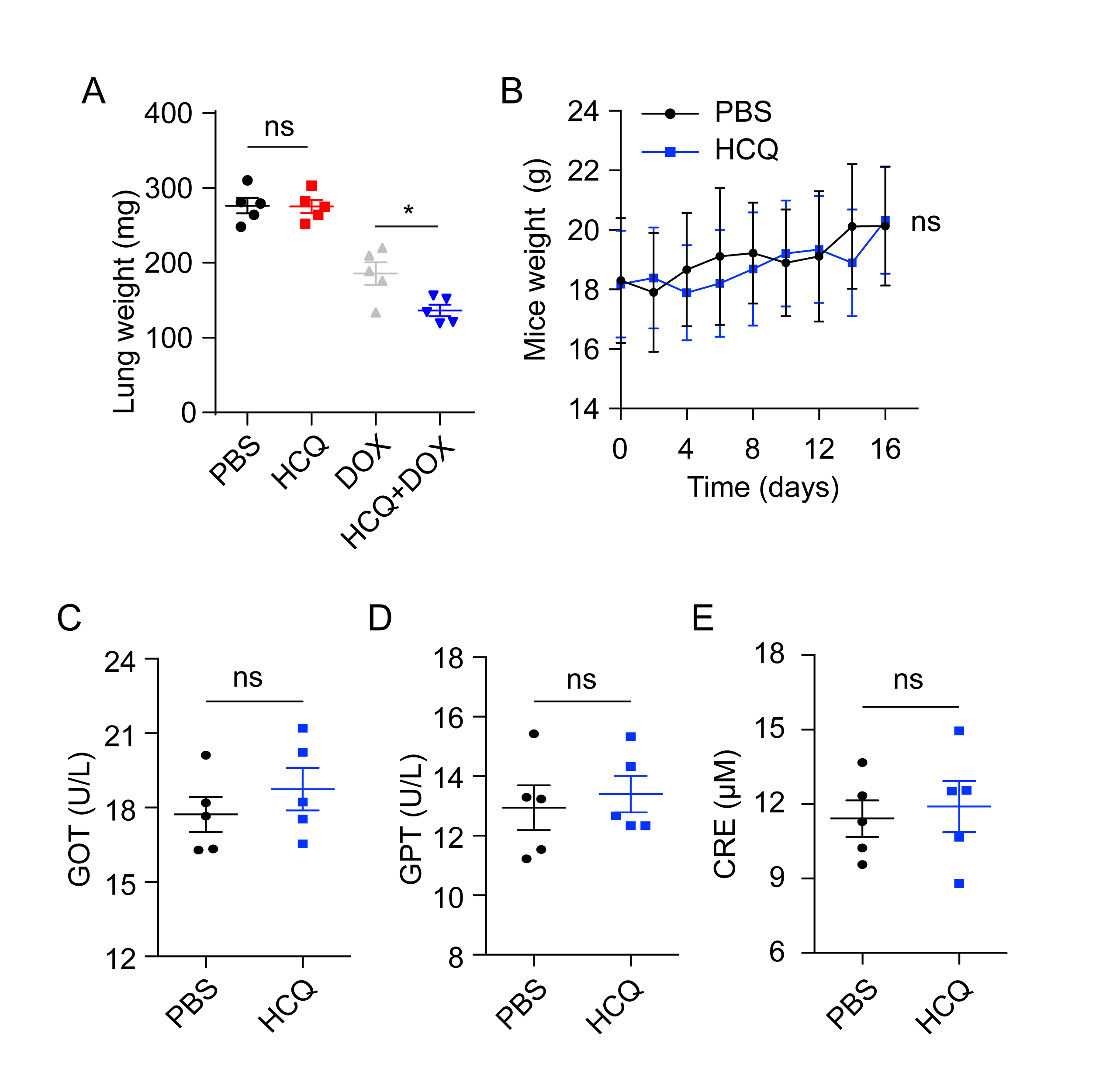

Supplement: Supplementary file 1 — Figure S1. (A) A total of 2 × 10^6^ A549 cells were intratracheally instilled into the lungs of nude mice. On day 20, the mice were instilled with 10 mg/kg HCQ and then followed with 2 mg/kg DOX treatment by tail vein injection every 2 days. Mice were treated for a week. After 2 weeks, mice were sacrificed for lung weight evaluation. (B, C, D, E) Female C57BL/6 J mice were treated with 10 mg/kg HCQ. The mice were sacrificed on day 3 for the systemic toxicity analysis. (B) Weights of the mice in each group. (C) Glutamic-oxaloacetic transaminase (GOT) detection in each group. (D) Glutamic-pyruvic transaminase (GPT) detection in each group. (E) Creatinine (CRE) detection in each group. For all graphs, the error bars indicate the mean ± s.e.m., *P < 0.05; **P < 0.01; ***P < 0.001; ns, no significant difference. The data shown are representative of three independent experiments. (TIF 1095 kb) [file 13046_2018_938_MOESM1_ESM.tif]

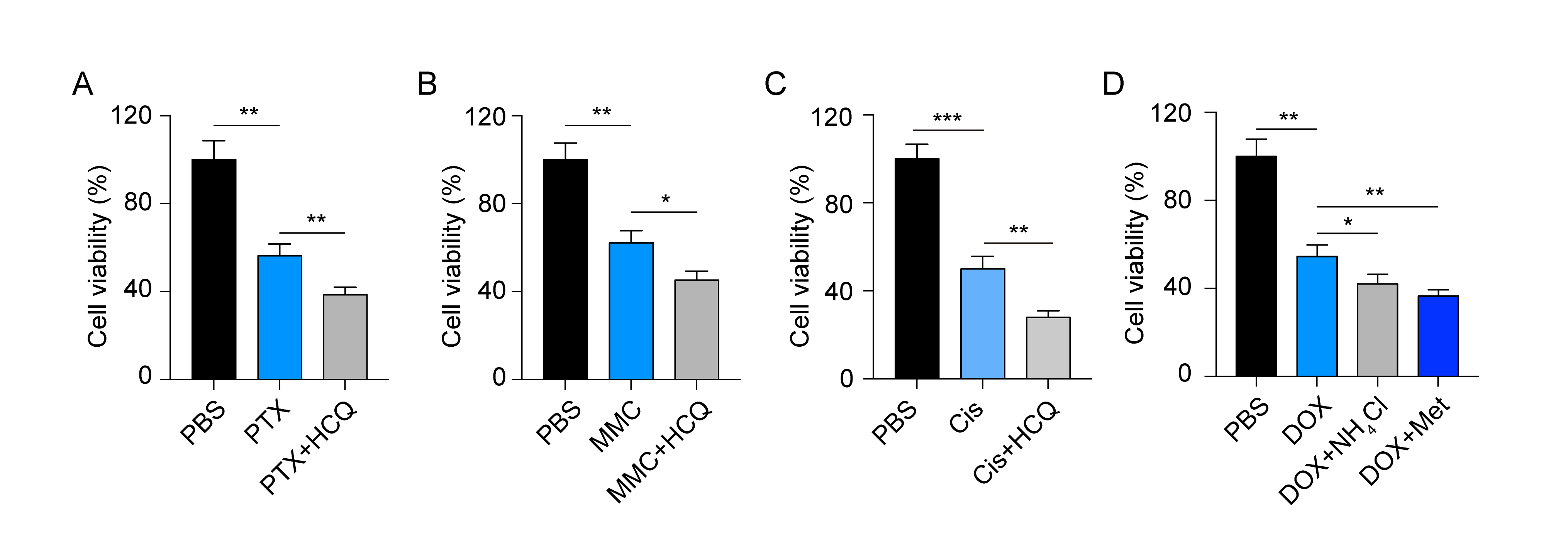

Supplement: Supplementary file 2 — Figure S2. (A) Lewis cells were pre-treated with PBS or 5 μM HCQ and followed by 0.5 μg/mL PTX treatment for 24 h. Apoptosis was detected by MTT analysis. (B) Lewis cells were pre-treated with PBS or 5 μM HCQ and followed by 1 μg/mL MMC treatment for 24 h. Apoptosis was detected by MTT analysis. (C) Lewis cells were pre-treated with PBS or 5 μM HCQ and followed by 0.5 μg/mL Cis treatment for 24 h. Apoptosis was detected by MTT analysis. (D) A549 cells were treated with 1 μM NH4Cl or 1 μM Met and followed by 0.5 μg/mL DOX treatment for 24 h. Apoptosis was detected by MTT analysis. For all graphs, the error bars indicate the mean ± s.e.m., *P < 0.05; **P < 0.01; ***P < 0.001; ns, no significant difference. The data shown are representative of three independent experiments. (TIF 953 kb) [file 13046_2018_938_MOESM2_ESM.tif]

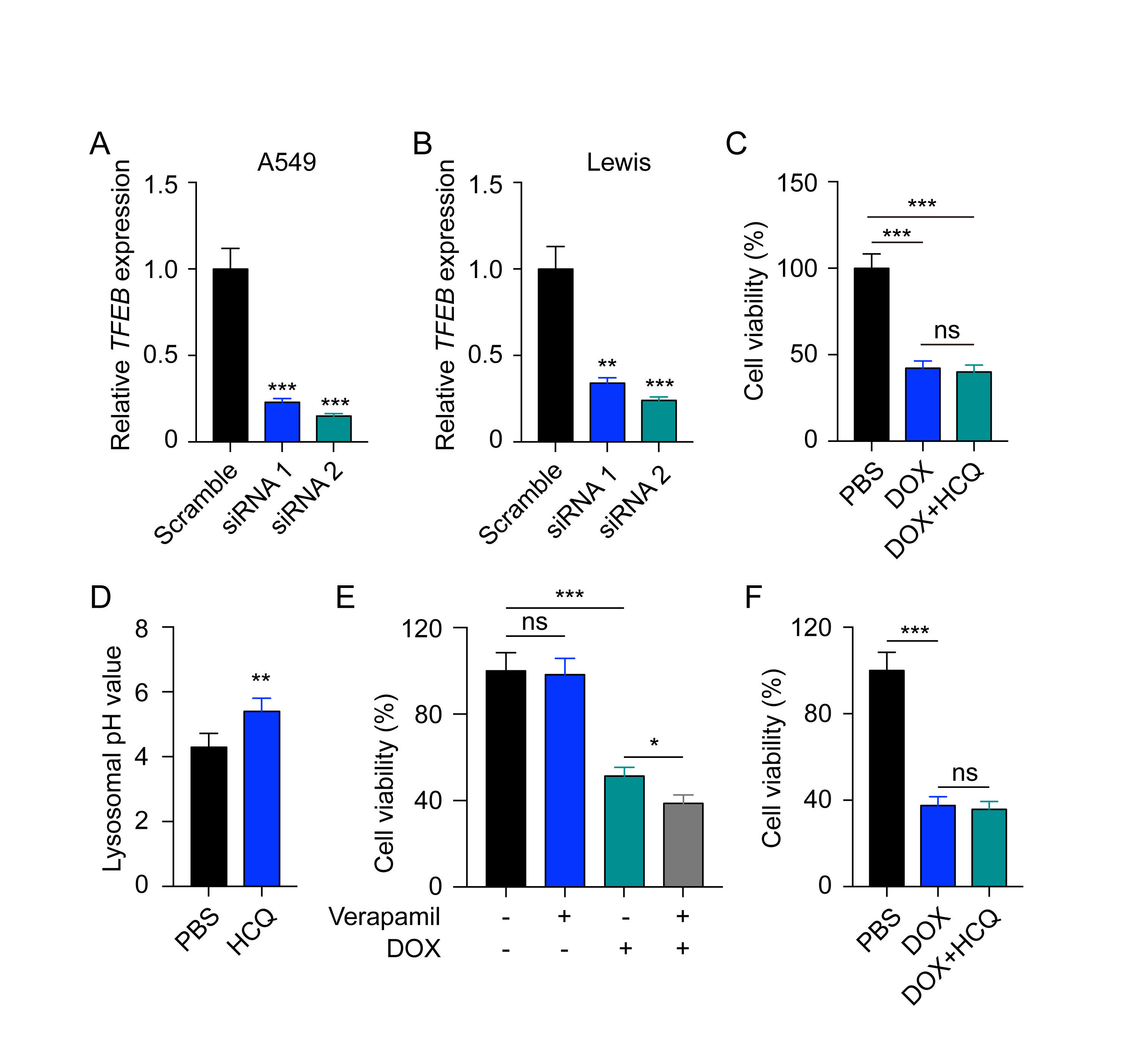

Supplement: Supplementary file 3 — Figure S3. (A) Relative TFEB expression in mRNA levels of A549 cells treated with TFEB siRNA. (B) Relative TFEB expression in mRNA levels of Lewis cells treated with TFEB siRNA. (C) The viability of TFEB-silenced Lewis cells treated with DOX or 0.5 μg/mL DOX combined with 5 μM HCQ for 24 h. (D) The lysosomal pH values of Lewis cells treated with PBS or HCQ (5 μM) for 12 h. (E) The viability of verapamil-pre-treated A549 cells treated with 0.5 μg/mL DOX for 24 h. (F) The viability of verapamil-pre-treated A549 cells treated with PBS, 0.5 μg/mL DOX or DOX combined with 5 μM HCQ for 24 h. For all graphs, the error bars indicate the mean ± s.e.m., *P < 0.05; **P < 0.01; ***P < 0.001; ns, no significant difference. The data shown are representative of three independent experiments. (TIF 1530 kb) [file 13046_2018_938_MOESM3_ESM.tif]

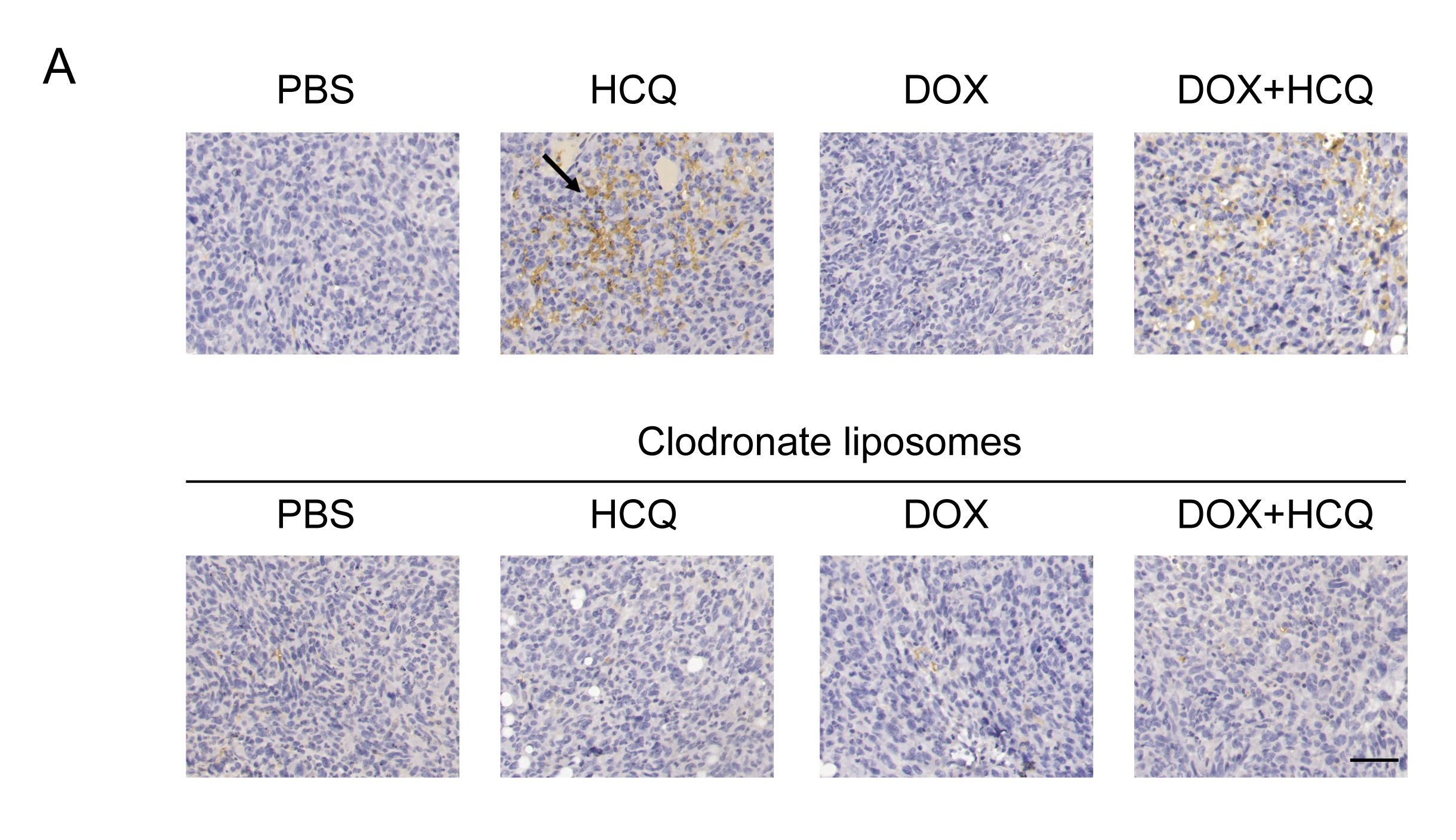

Supplement: Supplementary file 4 — Figure S4. (A) Lewis-bearing C57BL/6 J mice (n = 5) were instilled with HCQ and/or i.v. followed by i.v. DOX treatment in the presence or absence of clodronate liposomes. On day 24, mice were sacrificed and tumour tissues were fixed for immunohistochemistry. The represented pictures showed the CD8 expression of lung tumour tissues in each group (scale bar, 50 μm). (TIF 7658 kb) [file 13046_2018_938_MOESM4_ESM.tif]
